# Medical Observations during a Tour through Europe in 1875–’76

**Published:** 1876-09

**Authors:** Swan M. Burnett


### SOUTHERN MEDICAL RECORD.

VOL. VI. SEPTEMBER, 1876. NO. 9

### Original Communications.

### MEDICAL OBSERVATIONS DURING A TOUR THROUGH EUROPE IN 1875–'76.

BY SWAN M. BURNETT.

(Read, by request, before the Knox county section of the East Tennessee Medical Society, and ordered to be published in the SOUTHERN MEDICAL RECORD.)

GENTLEMEN: The remarks which I will make, according to your request, this evening, will be rather gossippy in their nature, the consideration of weightier and more scientific matters being deferred to another season.

The relations which medical men bear to each other and to the community are not the same in any two of the countries it has been my fortune to visit. There is more uniformity in this particular among the different nations on the continent, but they are all essentially different from those relations as found in England, which in all matters, social and political, is a law unto itself, and desires to be, and must be, judged by no standard but its own. No country that I have visited clings to customs because they are old, or rejects them because they are new, to the extent that it does. Conservatism within proper bounds is eminently commendable; and in an inexact science, such as medicine, anything like an overreaching radicalism is to be highly deprecated. But when we allow conservatism to degen erate into stupidity, we not only clog the wheels of progress, but allow ourselves to fall into the liability of doing a positive harm, since "*qui stat, retro stat.*" I do not mean to say by this that there are no progress.ve men in England. Very far from it. You all know what some Englishmen have done for medicine as an art. But I mean that, as a rule, you will find them steady-going, easy, satisfied practitioners, content with their present knowledge, and with but little inclination to take hold of any new-fangled ideas—especially if those new-fangled ideas come from abroad. The fight that British surgeons, as a rule, made against the use of isidectony in glaucoma is a shame to the country that has held such men as McKenzie and Bowman· Of course, they have had to give in, and now no people are louder in their laudations of the remedy they once condemned; but can a people so loath to seize upon new facts, or accept new truths, ever lead the vanguard of science?

The Museum of the Royal London Ophthalmic Hospital offers more material for the study of the pathology of the eye than any other institution in existence; but to what country and what institutions do we look for the most advanced knowledge of the pathological histology of the eye? To the small university towns of Germany, where the scarcity of material is compensated for by persistent labor and patient investigation.

As a rule, too, (though this is something many other countries have in common with England), English physicians and surgeons are more or less intolerant of foreign practitioners who settle among them. And yet it is remarkable that the man who is reported as doing the largest ophthalmic practice in London, and as receiving the largest income, is a German, who, though deemed quite clever, is yet by no means attractive in his person or agreeable in his manners.

The greatest fault I have to find with the Hospitals (I speak now only of the ophthalmic) is the lack of system or concert of action in the matter of teaching. The material, as I have said, is immense, but it is so much scattered among the different surgeons, and the time is so limited, that it is impossible for the student to derive the full benefit from the advantages that are to be had. Moorfileds could be made the great ophthalmological school of the world.

But when you leave the generals and come down to particulars, the aspect of matters is materially changed. The surgeons are all gentlemen who receive you, though a stranger, courteously, and treat you kindly and allow you the utmost freedom in the uses of their cases for examination. In their intercourse with hospital patients, too, they are kind and considerate to a degree rarely seen in Germany or among German surgeons anywhere.

One reason why English surgeons have done and are doing so little for the advance of medicine, as a science, is that, if they are inclined to scientific pursuits in their younger days, as soon as practice begins to come in they must forsake their investigation to attend to their patients. Private practice is the great end to be attained by the medical man in England. There are few men indeed who are willing to give themselves up wholly to scientific labors simple and pure; and, strange as it may seem, a scientific reputation is a bar rather than a help to the attainment of private practice. The practice of medicine is still divided up into physicians and surgeons and general practitioners, as it has been for decades of years. The customs of English society are as unalterable as the laws of the Medes and Persians, and woe to the medical man who dares to transgress them.

The general practitioner is the man who does everthing, including midwifery, and corresponds to the same individual with us. He makes up his own medicines (or has an assistant who does it for him) and regulates his prices by the capacity of the individual to pay, though, as a rule, they are fixed by the custom of the neighborhood in which he lives. He is allowed to have a sign, and can make it as large and conspicuous as he pleases. He usually styles himself "Surgeon," and commonly intimates in his window that "midwifery cases are attended." His style of living is not of so much moment as that of a "consultant," but he must conform strictly to the manner of living adopted by the community in which he resides.

As regards the other two classes—the consulting physician and surgeon—the case is different. They are never supposed to see a case, except it be sent by a medical man, and the fee must never be lower than a guinea. He must live within certain prescribed limits (at present in London the orthodox locality is in the neighborhood of Cavendish Square), and his rental must not fall below a certain sum, and there are certain prescribed outward appearances which are as essential as his fellowship of the College of Physicians or Surgeons. He is allowed no sign, the utmost that is admissable being his name on the door-plate. If he expects to occupy a good position, he must have an appointment to some hospital. That, you may say, is indispensable. And as the number of those places, even in a large city like London, is necessarily limited, and the number of applicants almost unlimited, the struggle for a place, when it becomes vacant, is very active, and, as is the case with us, personal influence is more often successful than professional merit.

The life of a young consultant in London is often a wearisome business indeed. At least ten years of waiting are before him, and often many more. I know men in London—able and capable men, who have worked and waited until they are forty, who do not see four private patients a month. I think it is a matter of greater difficulty to get established in a consulting practice in London than in any other city on the globe.

The division of consultants into physicians and surgeons, exists now as it did a hundred years ago. As you are aware, the surgeon is still called Mr., though many of them are M. D's. A surgeon cannot act as a physician, nor can a physician perform the duties of a surgeon. The subdivision of surgery into specialties, however, is not carried on to the extent it is here or in Germany. There are not more than eight or ten men in London who devote themselves exclusively to ophthalmology, and a surgeon never adopts a specialty until he is forced to. Mr. Bowman never gave up general surgery until his increase of ophthalmic practice gave him no time to attend to other matters. Mr. Hutchinson still does a large practice in general surgery, and is the Senior Surgeon to the London Hospital.

The general practitioner is seldom an M. D. He is generally a licentiate of some of the various institutions that are empowered to grant permission to practice.

In France an entirely different *regime* prevails. No man can practice without a special license unless he is a Doctor in Medicine of the Faculty of Medicine of Paris, or one of the other universities of France. There is no country in the world that is so hard on quacks, or that supports the regular profession to the extent that France does, and nowhere can a medical education, so far as the acquisition of knowledge is concerned, be obtained so cheaply as in Paris. In England a medical education cannot be obtained for less than three or four thousand dollars. In Paris a most thorough education in all the branches of medicine can be had without the expenditure of a *sou*, except a trifle for the material used in the laboratories. In the *Laboratoire d'Histologie* you have a microscope and all the accessories and materials and instruction for five francs (one dollar) per month.

In France every practitioner, be he a physician or surgeon, is a doctor, and there is no general division into general practitioners and consultants, though of course there are men who do nothing but a consulting practice. Most of the specialists depend largely upon their medical brethren for cases, but when a man has once attained to a prominent position he becomes so well known among the people as to be, to a large extent, independent of their aid.

The French people are exceedingly generous toward foreigners who settle among them. In fact, some of their most prominent men are of foreign birth and education. Wecker, the leading ophthalmologist, is a German, and you all know the success awarded to Sims and Brown-Seguard. No country is so liberal in the rewarding of true merit, irrespective of the nationality of the individual, as France.

The French doctor seldom has a sign or any token by which you can find his residence, and he frequently has his office on the third floor.

Private institutions, or *maisons de sante*, are much in vogue, among the specialists in particular, and all the leading ophthalmologists have them. For, strange as it may seem, notwithstanding the liberality of the French government, which in most respects is unequaled, the School of Medicine has never yet been prevailed upon to establish a chiar of ophthalmology. All the ophthalmological teaching that amounts to anything is done in those private cliniques, such as are common in Germany, and of which we have two or three examples in America. In England such a thing is unheard of, and woe betide the man who should endeavor to establish one. In some particulars this private clinique system is an admirable one. It is, as you can suppose, an expensive one to the beginner, and even at its best it but poorly repays the outlay necessary for its support, but all this demands an enthusiasm for the carrying forward that cannot but redound to the good of the science. These cliniques are advertised in a way, too, that would not be permitted either in England or America. Large posters are to be seen on all the walls, such as the following:

CLINIQUE FOR DISEASES OF THE EYES!

THE COURSE FOR DISEASES OF THE EYE BY DR. ——— WILL BEGIN ON THE 1ST OF NOVEMBER AND BE CONTINUED EVERY TUESDAY AND FRIDAY THEREAFTER AT $1\frac{1}{2}$ O'CLOCK.—THE POOR CAN OBTAIN ADVICE GRATIS.

Sometimes it is stated that a book on diseases of the eye by the proprietor of the clinique has just been issued at a certain price. Another plan the French author has of making a *reclame*, as he calls it, is to have printed on the fly-leaf of any *brouchure* he may publish a list of "publications by the same author." All these little irregular modes (according to our manner of thinking) are at least tolerated since there is so little of the kind of quackery there that we have to contend with in this country.

The school of medicine not recognizing ophthalmology as a distinct branch of Surgery, ophthalmologists are forced to do something for their own protection, and for the advancement of their favorite science. Almost every hospital surgeon does opthalmic surgery as well, and in the wards of almost any general hospital you will see cataract extractions, iridectonies, enucleations, and other operations on the eye and its appendages. The non-recognition of ophthalmology as a distinct branch of surgery, is a bit of poor economy, to say nothing of anything else. It has been estimated that the average number of days of "keep" of a cataract extraction in a general hospital is three times as many as in one of the private cliniques. The reasons of this, are that the operations are not so skillfully performed, and the special arrangements for nursing and care in after treatment are wanting.

The natural politeness of a Frenchman does not forsake him in his clinique. To all he is kind and attentive, and the universal expressions of thanks and gratitude on the part of his patients must certainly add much to the pleasantness of his task, and lighten in no small degree the fatigues of his labors. His consultation room is neat and tasteful in its arrangements, and the patients are, with rare exceptions, clean and orderly.

In France they seem to have settled the question as to the proprietorship of the prescription. It is always retained by the patient. The apothecary makes a copy of it in a book he keeps for the purpose, and having stamped the original with the name of his house, returns it to the patient.

My observations in Germany were not so extended as in either France or England. I spent several days at the clinique of Dr. Mooren, at Dusseldorf—the largest single one in Prussia, not even excepting Berlin. Some peculiarities in this institution deserve mention. His clinique is also his private consultation room. Here he receives all his patients from the princes to the beggar.

In the strict acceptation of the term, his cannot be said to be a free clinique. Every one, except the most extreme poor, are expected to pay something for advice. Generally, the amount charged was very trifling—ten silbergroschen (twenty-four cents). The largest sum I saw paid by the apparently well-to-do was one thaler (seventy cents).

This, it appears to me, is a move in the right direction, and a step towards the solution of the question, "What shall we do with our sick poor?" The abuse of free hospitals in Europe is as great as it is in this country, if not greater, and the question above quoted must before long present itself to us for action, and now is the time to prepare for it and not wait as Europe has, till custom has become crystalized into fact, and cannot be changed. The rights of the physician and of the community which contributes to the support of charitable institutions, demand that the abuses of our hospital system be stopped, and some modification of the plan just mentioned seems to me to be the best calculated to meet the requirements of all parties.

The clinique of Dr. Mooren is not so neat as those of Paris, or of his neighbors at Utrecht in Holland.

In Holland the hospitals, as well as everything else, are as clean as water can make them. Utrecht is the residence of the immortal Donders, the man who has not only achieved a grand success in general physiology, but has done more than any other man to bring into practical utility the laws of physiological optics. His work on Refraction and Accommodation is classic, and will remain so as long as ophthalomogy is a science. The clinique, under charge of him and his associate, Dr. Snellen, is not large, but its material is used to the very best advantage. The private office of Donders and Snellen is the best arrranged for the purpose of any I have seen.

In Italy I saw but little of medicine. Dr. Mannhart, who lives in Florence, was not in the city at the time I was there. Prof. Schiff also lives in Florence, and I made arrangements to visit him in his laboratory in accordance with an invitation sent through a mutual friend, but unforseen circumstances prevented my going. I very much regret this, for Prof. Schiff is as learned a physiologist as is now living. In practice, he devotes himself mostly to diseases of the nervous system. It was against him, as some of you will remember, that the battle against vivisection first began to be waged.

There are many other points I would like to touch upon, but my paper has already outgrown its proper limits, and in conclusion I can only say that if any of you should chance to make he same tour, I trust you may meet with the same kindness as I did; and I am sure you will, for the statement that you are an American physician is all the letter of introduction you need to secure anywhere in Europe all the civilities due from one professional brother to another.

